# Supplementary material for: Global water gaps under future warming levels
Source: Nat Commun. 2025 Jan 30;16:1192. doi: 10.1038/s41467-025-56517-2 (PMC11782649; doi:10.1038/s41467-025-56517-2)
Supplement: Supplementary file 1 — Supplementary Information [file 41467_2025_56517_MOESM1_ESM.pdf]

Supplementary Information for

Global water gaps under future warming levels

Authors: Lorenzo Rosa[1,*] and Matteo Sangiorgio[2]

Affiliations:

[1] Biosphere Sciences and Engineering, Carnegie Institution for Science, Stanford, CA, 94035, United States of America

[2] Department of Electronics, Information and Bioengineering, Politecnico di Milano, Via Ponzio 34/5, 20133, Milan, Italy

***Corresponding author:** lrosa@carnegiescience.edu

This file includes:

- Supplementary Tables 1 and 2
- Supplementary Figures 1 to 7

Supplementary Table 1. The 40 countries with the largest water gaps worldwide. Water gaps are presented under baseline, 1.5°C, and 3°C warmer climate. See Supplementary Dataset for detailed model-specific results.

km^3 per year		**1.5°C warming**			**3°C warming**		
	**Baseline**	**Max**	**Min**	**Average**	**Max**	**Min**	**Average**
India	124.3	154.1	118.9	135.4	160.7	125.1	141.4
United States	53.8	59.1	54.4	56.1	69.9	52.8	61.2
Pakistan	35.8	44.0	32.1	38.2	67.1	36.6	47.5
Iran	35.0	38.2	33.9	35.8	37.9	35.0	37.0
China	27.2	37.8	22.5	31.3	37.0	31.2	34.5
Iraq	23.8	25.4	23.1	24.3	26.4	24.1	25.1
Egypt	18.8	19.3	18.2	18.7	19.5	18.5	19.0
Uzbekistan	16.6	17.3	15.6	16.5	17.1	15.6	16.4
Saudi Arabia	12.1	12.4	11.6	11.8	16.9	12.2	13.5
Turkmenistan	11.1	11.4	10.3	10.9	11.9	10.6	11.3
Mexico	9.6	9.9	8.3	8.9	11.7	8.9	10.4
Turkiye	6.9	8.7	6.6	7.9	9.8	8.1	9.0
Chile	6.6	6.9	6.5	6.7	7.5	6.8	7.1
Vietnam	6.4	7.3	4.9	5.9	6.9	5.2	6.1
Afghanistan	6.1	6.5	5.4	6.1	7.5	5.8	6.7
Spain	5.7	7.9	6.5	7.3	10.6	7.5	9.2
Sudan	4.6	4.5	3.5	4.1	4.5	3.6	4.0
Syria	3.9	4.5	3.7	4.0	4.7	3.9	4.3
Kazakhstan	3.5	3.8	3.0	3.5	4.0	2.9	3.4
Bangladesh	3.1	3.5	2.7	3.2	7.0	2.6	4.4
Morocco	3.0	4.0	3.2	3.7	4.5	3.8	4.3
Argentina	2.7	3.2	2.7	3.0	3.6	2.8	3.2
Philippines	2.4	2.6	1.7	2.0	2.7	1.8	2.3
United Arab Emirates	2.1	2.1	2.1	2.1	2.2	2.1	2.1
Indonesia	1.9	3.4	0.8	2.0	3.8	1.3	2.1
South Africa	1.9	2.7	1.8	2.2	2.7	1.7	2.4
Libya	1.7	1.9	1.5	1.7	2.1	1.8	1.9
Tajikistan	1.6	1.7	1.5	1.6	1.6	1.4	1.5
Kyrgyzstan	1.5	1.8	1.2	1.6	2.0	1.1	1.5
Greece	1.5	2.3	1.5	1.8	2.7	1.7	2.1
Thailand	1.4	1.8	1.3	1.5	2.4	1.1	1.7
Portugal	1.3	1.5	1.2	1.4	2.1	1.3	1.7
Azerbaijan	1.2	1.5	1.2	1.4	1.6	1.4	1.5
Nigeria	1.2	1.2	0.4	0.8	0.9	0.4	0.6
Australia	1.0	1.8	1.1	1.5	2.1	1.7	1.9
Peru	1.0	1.4	0.9	1.0	1.2	0.7	1.0
Italy	1.0	2.0	1.0	1.4	2.6	1.5	2.3
Canada	0.9	1.2	0.6	0.9	1.3	0.8	1.0
Myanmar	0.9	1.1	0.8	1.0	1.4	0.9	1.1
Russian Federation	0.9	1.0	0.8	0.9	1.4	0.8	1.1

Supplementary Table 2. The 40 major hydrological basins with the largest water gaps worldwide. Water gaps are presented under baseline, 1.5°C, and 3°C warmer climate. See Supplementary Dataset for detailed model-specific results.

km^3 per year		**1.5°C warming**			**3°C warming**		
	**Baseline**	**Max**	**Min**	**Average**	**Max**	**Min**	**Average**
Ganges - Bramaputra	56.1	74.1	53.5	61.7	80.8	55.0	67.9
Sabarmati	52.6	55.2	47.7	51.2	60.9	48.4	54.2
Tigris - Euphrates	34.1	37.5	33.3	35.1	38.1	35.3	36.7
Indus	28.7	33.5	25.2	30.2	49.7	30.0	37.1
Nile	22.2	22.5	20.5	21.6	22.2	20.9	21.5
Mississippi - Missouri	17.1	22.8	17.2	19.6	28.5	17.1	22.6
Caspian Sea, East Coast	16.6	17.3	15.3	16.5	18.0	15.9	17.1
Central Iran	14.3	15.3	13.9	14.5	15.3	14.1	14.7
Amu Darya	14.2	14.8	13.2	14.1	15.0	13.5	14.4
Columbia and Northwestern United States	13.2	13.9	11.4	12.6	13.5	12.1	12.9
California	12.8	13.3	12.1	12.9	14.0	11.2	13.0
Arabian Peninsula	11.9	11.9	11.3	11.5	16.6	11.9	13.2
Krishna	10.9	14.3	9.9	12.2	12.4	10.8	11.8
Syr Darya	9.9	10.7	8.9	9.9	10.5	8.6	9.7
Ziya He, Interior	8.5	11.1	5.5	8.6	15.3	6.5	10.2
Caspian Sea, South West Coast	7.7	8.8	7.7	8.3	9.1	8.7	8.9
Godavari	5.3	10.1	6.5	7.8	9.0	5.3	8.0
Mediterranean Sea, East Coast	5.3	6.3	5.2	5.8	6.9	6.0	6.5
China Coast	5.2	10.5	3.9	7.2	10.2	5.6	7.9
Mexico, Northwest Coast	4.8	4.7	4.0	4.4	5.3	4.1	4.9
Huang He	4.7	6.8	3.7	4.9	5.9	4.3	5.1
Mekong	4.4	5.0	3.0	4.1	5.4	3.7	4.4
North America, Colorado	3.8	3.8	3.1	3.5	4.1	3.4	3.6
Persian Gulf Coast	3.4	3.5	3.1	3.3	3.8	3.3	3.5
South Chile, Pacific Coast	3.3	3.5	3.2	3.3	3.9	3.4	3.6
North Chile, Pacific Coast	3.2	3.4	3.2	3.3	3.5	3.3	3.4
Africa, North West Coast	3.1	4.0	3.3	3.8	4.5	3.9	4.3
Mediterranean South Coast	2.7	3.5	2.8	3.2	4.0	3.2	3.7
Gulf Coast	2.6	4.4	2.1	3.3	5.5	3.1	4.4
Helmand	2.6	2.7	2.4	2.6	3.1	2.3	2.8
Philippines	2.4	2.6	1.7	2.0	2.7	1.8	2.3
Great Basin	2.1	2.1	1.6	1.9	2.1	1.8	2.0
Red Sea, East Coast	2.1	2.9	2.0	2.4	2.8	2.2	2.5
Gobi Interior	2.1	2.6	1.8	2.2	2.6	1.7	2.2
Eastern Jordan - Syria	2.1	2.2	2.0	2.1	2.2	2.1	2.2
Tarim Interior	2.1	3.2	2.1	2.4	3.6	2.0	2.5
Bo Hai - Korean Bay, North Coast	2.0	3.1	1.6	2.3	3.0	1.7	2.4
Java - Timor	1.9	3.3	0.8	2.0	3.7	1.3	2.1
Rio Grande - Bravo	1.8	1.8	1.3	1.7	2.2	1.5	1.9
Guadalquivir	1.7	2.2	1.9	2.1	3.1	2.2	2.7

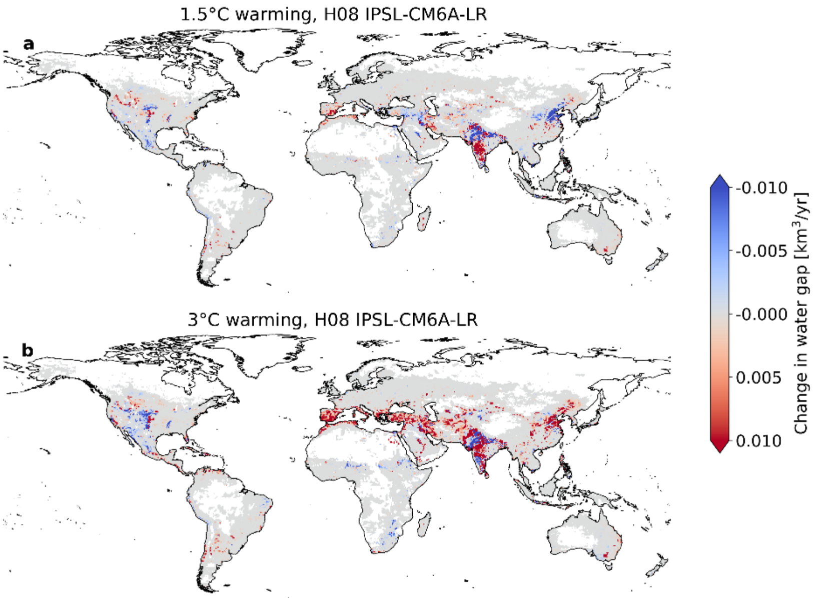

Supplementary Figure 1. Change in water gap with IPSL-CM6A-LR for 1.5°C (a) and 3°C (b) warming climate with respect to the baseline (2001-2010). Values are in km^3/yr per pixel. Pixel resolution is 0.5 arcminutes or 50 km at the Equator.

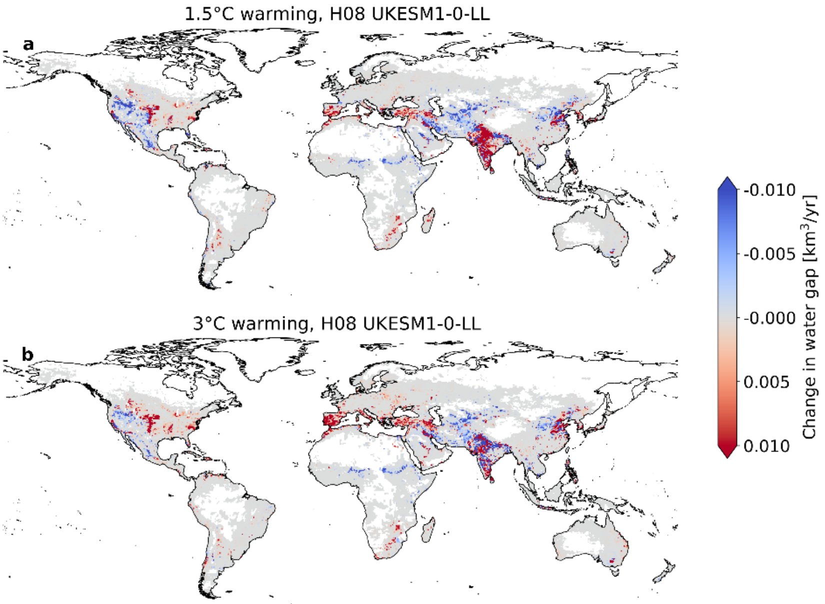

Supplementary Figure 2. Change in water gap with UKESM1-0-LL for 1.5°C (a) and 3°C (b) warming climate with respect to the baseline (2001-2010). Values are in km^3/yr per pixel. Pixel resolution is 0.5 arcminutes or 50 km at the Equator.

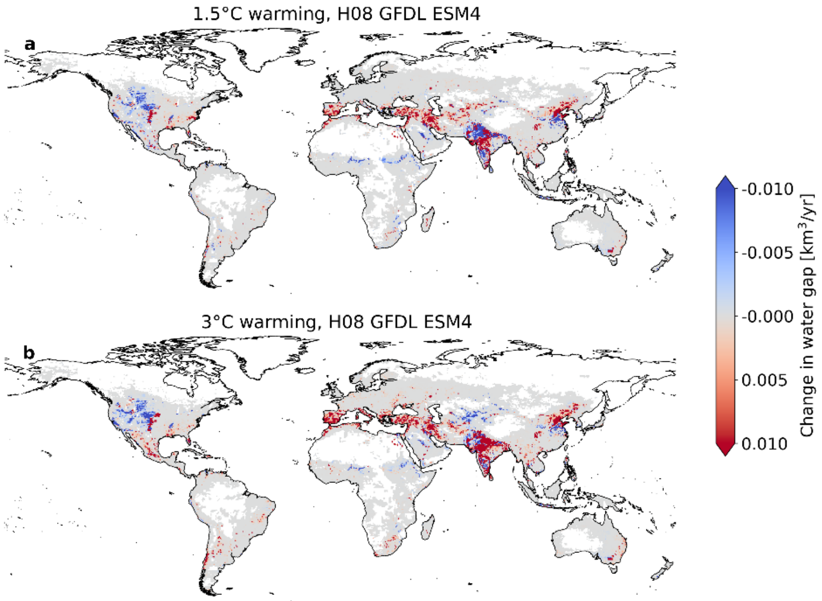

Supplementary Figure 3. Change in water gap with GFDL ESM4 for 1.5°C (a) and 3°C (b) warming climate with respect to the baseline (2001-2010). Values are in km^3/yr per pixel. Pixel resolution is 0.5 arcminutes or 50 km at the Equator.

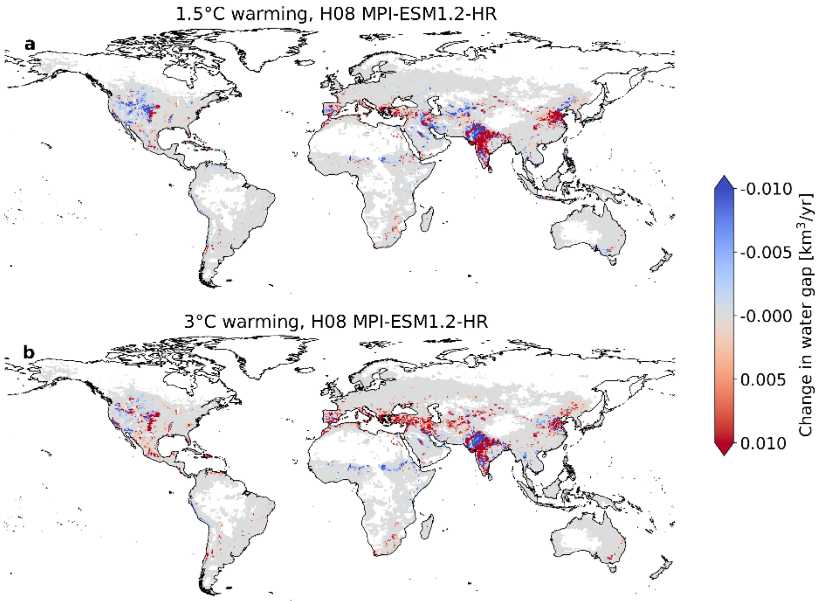

Supplementary Figure 4. Change in water gap with MPI-ESM1.2-HR for 1.5°C (a) and 3°C (b) warming climate with respect to the baseline (2001-2010). Values are in km^3/yr per pixel. Pixel resolution is 0.5 arcminutes or 50 km at the Equator.

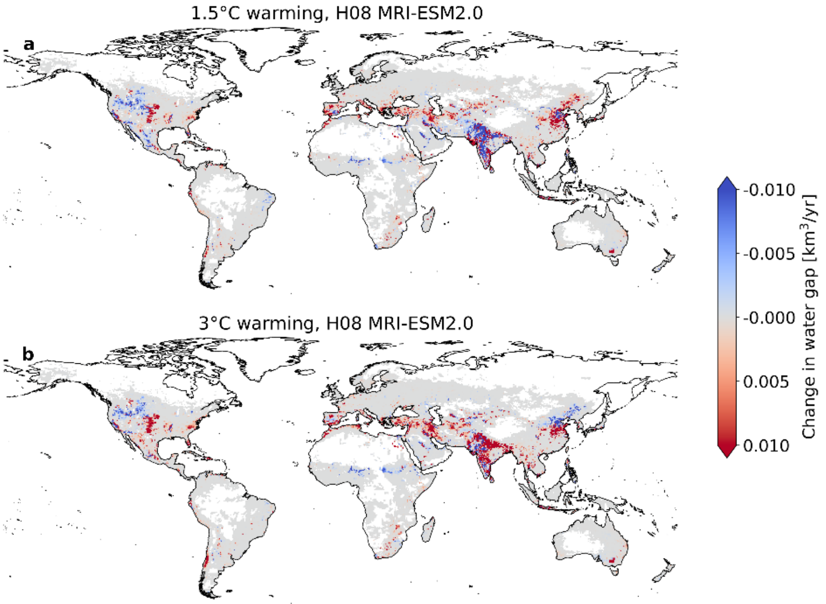

Supplementary Figure 5. Change in water gap with MRI-ESM2.0 for 1.5°C (a) and 3°C (b) warming climate with respect to the baseline (2001-2010). Values are in km^3/yr per pixel. Pixel resolution is 0.5 arcminutes or 50 km at the Equator.

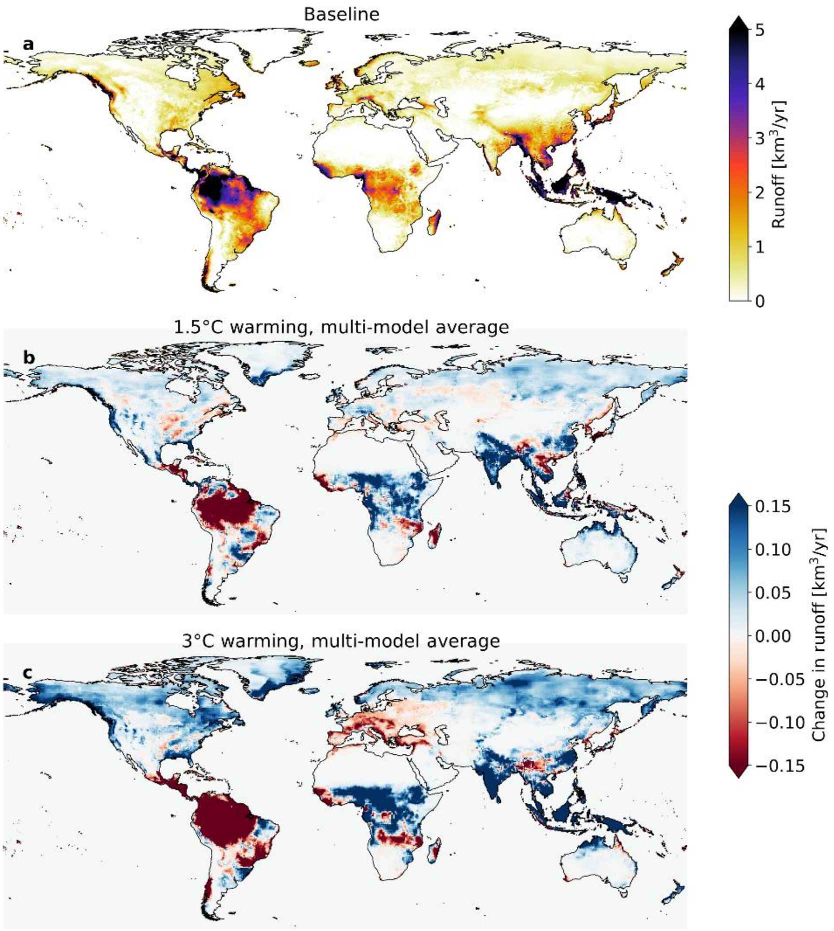

Supplementary Figure 6. Changes in runoff (surface and subsurface) under warming scenarios. (a) Runoff under baseline conditions. **(b, c)** Change in runoff under 1.5º C and 3º C warming. Values are in km^3/yr per pixel. Pixel resolution is 0.5 arcminutes or 50 km at the Equator.

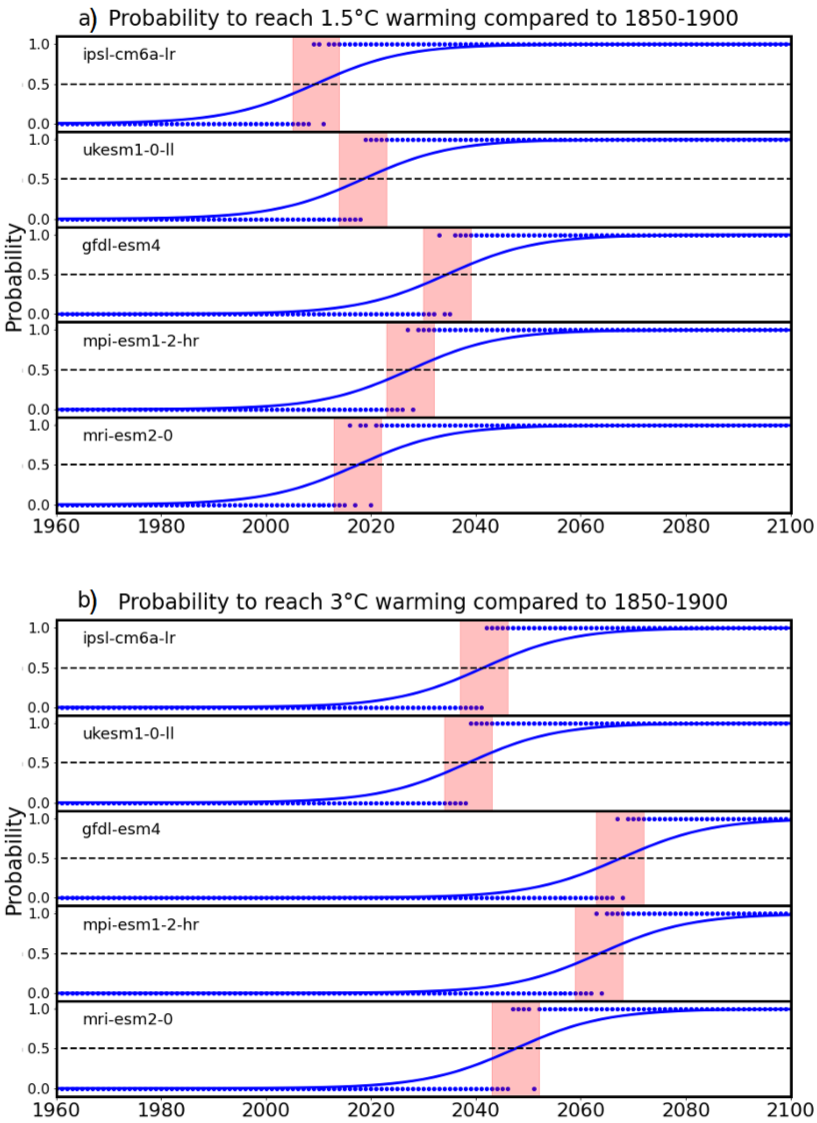

Supplementary Figure 7. The probability of each climate model reaching 1.5°C (a) and 3°C (b) warming in comparison to the pre-industrial era (1850-1900). The red area corresponds to the 10-year window within which this warming occurs. Based on: Beltran- Peña et al., 2024[1].

References

1. Beltran- Peña A, et al. Future Implications of Enhanced Hydroclimate Variability and Reduced Snowpack on California's Water Resources. ESS Open Archive. September 09, 2024. DOI: 10.22541/essoar.172589387.79380852/v1
